# Clinical Characteristics and Outcomes of COVID-19 in West Virginia

**DOI:** 10.3390/v13050835

**Published:** 2021-05-05

**Authors:** Sijin Wen, Apoorv Prasad, Kerri Freeland, Sanjiti Podury, Jenil Patel, Roshan Subedi, Erum Khan, Medha Tandon, Saurabh Kataria, Wesley Kimble, Shitiz Sriwastava

**Affiliations:** 1 Department of Biostatistics, West Virginia University, Morgantown, WV 26505, USA; siwen@hsc.wvu.edu (S.W.); keswails@mix.wvu.edu (K.F.); 2 Berkeley Medical Center, Department of Neurology, West Virginia University, Morgantown, WV 25401, USA; apoorvprasad@gmail.com; 3 Department of Medicine, Army College of Medical Sciences, New Delhi 110010, Delhi, India; sanjiti.1997@gmail.com; 4 Department of Epidemiology, Human Genetics and Environmental Sciences, The University of Texas Health Science Center at Houston (UTHealth) School of Public Health, Dallas, TX 75235, USA; Jenil.Patel@uth.tmc.edu; 5 Research Section, Nepal Health Research Council, Kathmandu 44600, Nepal; roshansubedi123@gmail.com; 6 Department of Medicine, B.J. Medical College and Civil Hospital, Ahmedabad 380016, Gujarat, India; erum2006@gmail.com; 7 Department of Neurology, University of Pittsburgh Medical Center, Pittsburgh, PA 15215, USA; medhatandon22@gmail.com; 8 Department of Neurology, Louisiana State University, Health Sciences Center, Shreveport, LA 71130, USA; saurabh.kataria27@gmail.com; 9 West Virginia Clinical and Translational Science Institute, Morgantown, WV 26505, USA; wkimble1@hsc.wvu.edu; 10 Department of Neurology, Rockefeller Neuroscience Institute, West Virginia University, Morgantown, WV 26505, USA

**Keywords:** COVID-19, SARS-CoV-2, mortality, severity

## Abstract

This study examines the clinical characteristics, outcomes and types of management in SARS-CoV-2 infected patients, in the hospitals affiliated with West Virginia University. We included patients from West Virginia with SARS-CoV-2 infection between 15 April to 30 December 2020. Descriptive analysis was performed to summarize the characteristics of patients. Regression analyses were performed to assess the association between baseline characteristics and outcomes. Of 1742 patients, the mean age was 47.5 years (±22.7) and 54% of patients were female. Only 459 patients (26.3%) reported at least one baseline symptom, of which shortness of breath was most common. More than half had at least one comorbidity, with hypertension being the most common. There were 131 severe cases (7.5%), and 84 patients (4.8%) died despite treatment. The mean overall length of hospital stay was 2.6 days (±6.9). Age, male sex, and comorbidities were independent predictors of outcomes. In this study of patients with SARS-CoV-2 infection from West Virginia, older patients with underlying co-morbidities had poor outcomes, and the in-hospital mortality was similar to the national average.

## 1. Introduction

The novel coronavirus disease 2019 (COVID-19) pandemic has caused widespread disruption of healthcare systems and economic calamity across the world [1]. Widespread equitable vaccination distribution efforts, all over the world, seem to be the only way out of this raging pandemic. Unfortunately, the United States has been an outlier among the developed countries severely affected by this pandemic with one in almost every five deaths worldwide being attributed to this virus [2]. The pandemic has exposed the fragile healthcare situation of the entire population and highlights the necessity of future mitigation efforts required to invest in the public health infrastructure of the country. 

An initial study by Chen et al. reported findings from 99 cases and suggested a higher incidence of infection in groups of older men with comorbidities coming in close contact, and also the development of acute respiratory distress syndrome (ARDS) [3]. Despite the widespread nature of the pandemic, most infected individuals showed a good prognosis with only mild to moderate clinical symptoms such as fever, non-productive cough, dyspnoea, myalgia, fatigue, and no evidence of pneumonia or pulmonary damage on routine imaging [4,5,6,7]. However, 20% of the reported cases showed a severe and progressive illness leading to ARDS, need for intubation, sepsis, and death [8,9,10,11,12,13]. 

With our increased understanding of the disease, it is essential to critically analyze and describe the clinical characteristics, treatment, and prognosis among different US subpopulations to derive relevant information regarding the course of the disease, disease management, as well as the long-term sequelae in survivors. Although certain epidemiological features and clinical characteristics of COVID-19 have been previously reported, [3,5,6] our study on a single-center (West Virginia University System hospitals) evaluates the clinical and demographic factors of COVID-19 patients, with regards to severity, mortality, length of stay and requirement of invasive lifesaving treatment. This data will be further helpful in predicting and possibly modeling the expected trajectory of the disease in patients.

## 2. Methods

### 2.1. Study Design

We conducted an observational study of inpatients with the diagnosis of COVID-19 between 15 April 2020 and 30 December 2020, in hospitals (Ruby Memorial hospital, Berkeley Medical Center, Camden Clark Medical Center) affiliated with West Virginia University (WVU). The study was approved by the Institutional Review Board (IRB) at WVU. All patients admitted or visiting the emergency room with established COVID-19 diagnosis were included and data were extracted from the hospital electronic medical records on 30 December 2020. The diagnosis was established with the identification of SARS-CoV-2 RNA by nasopharyngeal or oropharyngeal real-time RT-PCR (reverse-transcriptase polymerase chain reaction). 

### 2.2. Data Collection and Participant

Based on a standardized proforma, information regarding demographics (age, gender, ethnicity), clinical features (asymptomatic, cough, headache, fever, shortness of breath, respiratory distress, diarrhea, nausea/vomiting, cold/flu, fatigue, sore throat, body ache, and comorbid conditions: obesity, diabetes, hypertension), diagnosis (RT-PCR result), treatments (length of hospital stay in days, intubation, and type of medications received: hydroxychloroquine, azithromycin, remdesivir, tocilizumab), and outcomes (mortality) were collected for each included patient. The severity of COVID-19 was classified using Infectious Disease Society of America/American Thoracic Society (IDSA/ATS) criteria [14]. Length of stay was evaluated based on the duration of a single episode of hospitalization. Overall length of stay was calculated from the total number of episodes of hospitalization until the last follow-up. 

### 2.3. Statistical Analysis

Descriptive analysis was performed to assess the demographic and clinical characteristics, including proportions/percentages for categorical variables, and mean and median with standard deviations for continuous variables. Fisher’s exact test for categorical variables and the Wilcoxon rank-sum test for continuous variables were used to assess the difference in severity, mortality, and need for intubation among patient cohorts. Multivariable analysis was performed using logistic regression models to evaluate the association of demographic characteristics with clinical outcomes such as severity (yes/no) and mortality (yes/no) in COVID-19 patients. Final models were obtained based on the backward variable selection process. Finally, the Kaplan-Meier estimator and log-rank test were used to calculate event-free survival in which the event was defined as either death due to COVID-19 without severity or a severe COVID-19 diagnosis, whichever was reported first. All statistical tests were two-sided and a *p*-value of <0.05 was considered to be statistically significant. All data analysis was performed using statistical software R v3.6.3.

## 3. Results

### 3.1. Demographic Characteristics

A total of 1742 patients diagnosed with symptomatic COVID-19 were included in this study. The mean age of these patients was 47.5 (SD 22.7) years. The youngest patient was 5 months old and the oldest patient was 99.6 years old. Over half of the patients were less than fifty years of age (52.4%, *n* = 913) and were females (54%, *n* = 941). Majority of the participants were White (85%, *n* = 1472). 

### 3.2. Clinical Characteristics, Treatment, and Outcomes

Majority of the patients (73.7%) did not report any symptoms at the time of diagnosis. Of the reported symptoms, the most common symptom was shortness of breath (11.7%), followed by cough (4.1%) and fever (3.9%) (Table 1). Other symptoms reported by patients included cold/flu, sore throat, headache, fatigue, body aches, nausea/vomiting, and diarrhea. 

Over a half of patients (53.3%) had at least one comorbidity, of which hypertension was the most common (45.4%), followed by obesity (25.3%) and diabetes (25%). Almost all patients (98.3 %) were suspected to have COVID-19 before definitive diagnosis was established and the majority (96.5%) of them reported a history of exposure to SARS-CoV-2 before the onset of symptoms. Azithromycin was the most common drug used (9%) for treatment. There were 131 severe cases (7.5%), 124 patients (7.1%) requiring intubation, and 84 patients (4.8%) who died despite receiving treatment. Of the 84 deaths, 69 (18.96%) were in hospitalized patients and 15 (1.08%) were out of the hospital discharge or ER discharge patients. Table 1 shows further information on the clinical characteristics, treatments received, and outcomes in these patients.

The length of hospital stay for COVID-19 related first visits ranged from 0 to 108 days, with a mean duration of 2.2 days (SD 7.0). The duration of hospital stay in subsequent visits consistently decreased with a maximum of 9 days and a mean of 0.005 (SD 0.2) for a final fourth visit. The overall length of stay was 2.6(SD 7.9) with a maximum of 108 days of stay and a minimum of 0 days of stay in the hospital.

### 3.3. Analysis for Outcomes

We found that patients aged 50 years and above had a significantly higher occurrence of a severe infection [13% (109 patients) suffering from a severe infection as compared to 2% (22 patients) in younger group], *p* < 0.001. Moreover, older patients also showed a statistically significant higher intubation rate (12% in patients aged ≥50 years as compared to 2% in their younger counterparts), *p* < 0.001, and deaths (10% in patients aged ≥50 years, as compared to 1% in patients aged <50 years), *p* < 0.001 due to COVID-19, as compared to their younger counterparts (age < 50 years) (Table 2). 

Males had significantly more severe disease [9% compared to 6% in female patients (*p* = 0.023)], and were more likely to be intubated [9% compared to 6% in females, *p* = 0.031]. However, there was no significant difference in deaths between the two genders (*p* = 0.37). No statistically significant difference was noted for severity, fatality, and need for intubation between patients by race. 

A significant difference (*p* = 0.019) was noted in terms of severity of SARS-CoV-2 infection among patients who reported having a cough as compared to those that did not. Having a shortness of breath was significantly associated with the need for intubation (*p* < 0.001), high severity of COVID-19 (*p* < 0.001), and mortality (*p* = 0.008). A significantly higher number of patients who reported fatigue and diarrhea had a fatal outcome (*p* = 0.044 for fatigue and *p* = 0.03 for diarrhea). No statistically significant difference was observed for any of the other symptoms including cold, respiratory distress, fever, sore throat, headache, nausea, and vomiting. Hypertension and diabetes were significantly associated with intubation, severity, and mortality in the COVID-19 patients (*p* < 0.001), and obesity was associated with intubation and severity (*p* < 0.001).

The presentation with shortness of breath (5.8 days, *p* < 0.001), cough (2.7 days, *p* < 0.001), respiratory distress (5.5 days, *p* = 0.015), fever (4.0 days, *p* < 0.001), fatigue (6.2 days, *p* < 0.001), and nausea/vomiting (3.6 days, *p* < 0.001) resulted in significantly greater length of hospital stay for the first visit, as shown in Table 3. Although patients with diarrhea had the greatest number of hospital stay (mean = 6.3 days), the association was not significant (*p* = 0.073).

On comparing the severity of the disease amongst the various parameters under study, we found that age (*p* < 0.001), male sex (*p* = 0.007), shortness of breath (*p* = 0.024) and presence of comorbidities: hypertension (*p* = 0.004), diabetes (*p* = 0.004), obesity (*p* < 0.001) were independent predictors of severity in COVID-19 patients, as shown in Table 4. In terms of mortality, age (*p* < 0.001) and presence of hypertension (*p* = 0.018) were independent predictors of mortality. Moreover, we found significantly greater risk of disease severity among patients using Azithromycin (*p* = 0.02), Remdesivir (*p* = 0.02), and Tocilizumab (*p* < 0.0001). However, we found a greater risk of mortality among patients treated with Remdesivir (*p* = 0.002) and Tocilizumab (*p* = 0.05) (Table 4a,b).

**Figure 1:**
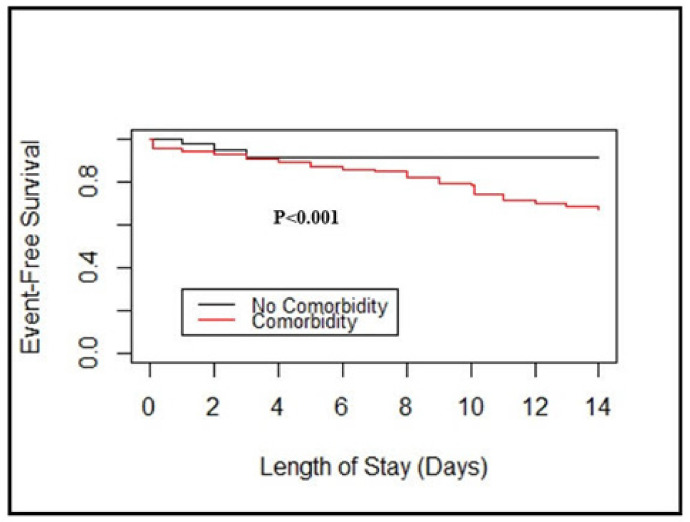
Event-free survival curves by comorbidity based on the Kaplan–Meier method. Statistical significance was calculated using a two-sided log-rank test.

**Figure 2:**
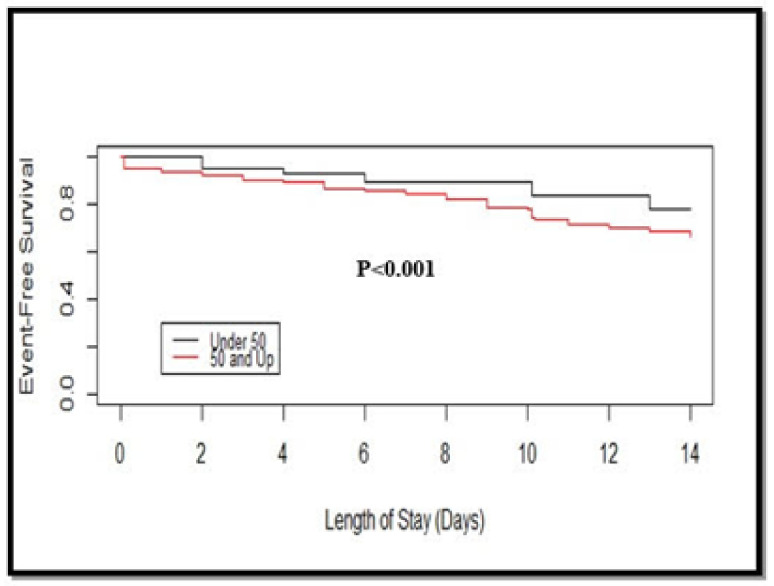
Event-free survival curves by age group (≤50 years or ≥50 years) based on the Kaplan–Meier method. Statistical significance was calculated using a two-sided log-rank test.

While studying the effect of comorbidities, the Kaplan-Meier method showed the difference in event-free survival between patients with or without the comorbidities (*p* < 0.001, log-rank test). There was a significant difference between patients aged < 50 years or aged ≥50 years (*p* < 0.001, log-rank test) in event-free survival analysis (Figure 1 and Figure 2). In particular, the 7-day event-free survival in patients with and without comorbidities was 84.6% and 91.3%, respectively; and the 7-day event-free survival was 84.3% for age ≥50 and 89% for age <50, respectively.

## 4. Discussion

The COVID-19 pandemic has disrupted healthcare, social and economic systems all over the world. Going into the pandemic, it was believed that West Virginia could face considerable challenges as it is highly populated with elderly populations. The fact that almost 40 percent of West Virginia’s population is rural, and 6.6 percent lack health insurance makes it the nation’s most vulnerable state bracing the impact of the COVID-19 pandemic [15,16,17]. Our study was conducted among the hospitals affiliated with West Virginia University that enrolled 1742 patients from 15 April 2020 to 30 December 2020, making it one of the largest representative samples from West Virginia. Consistent with the recent literature, we noticed a greater impact of COVID-19 among older adults of West Virginia [10,13,18]. 

The Centre for Disease Control (CDC) reports that patients aged more than 50 years are at a higher risk of developing severe infection (based on IDSA-ATS guidelines) and have a higher mortality rate than those aged less than 50 years [18]. This is attributed to the physiological changes that come with aging such as immunosenescence which alters pathogen recognition and clearing [19,20]. Our study concurs that patients aged more than 50 years had a higher occurrence of severe infection (*n* = 109 patients) as compared to patients aged less than 50 years (*n* = 22). Older COVID-19 patients had a higher rate of intubation (12%) as compared to the younger patients (2%) [21,22,23]. With respect to gender, males were more susceptible to severe infection which could be attributed to the elevated immune reactivity to viral infection in women as compared to men, due to enhanced antibody production which gives them an effective resistance to infection [24,25]. Our study showed a higher prevalence of severe COVID-19 infection in men (9%), compared to women (6%), and men were more likely to be intubated. However, our patient population was female predominant with 54% of the patients being females making the ratio of 1.17, in contrast to the male dominant patient demographics presented by other single-center reports [7,9,26,27].

In terms of ethnicity, most (*n* = 1472, 88.3%) of the patients were White, followed by others (*n* = 195, 11.7%) including Blacks, Asians, Hispanics, American natives, Pacific-Hawaii natives. This is slightly disproportional when the demographic make-up of the state of West Virginia is considered which is composed of Whites (93.5%), Blacks (3.6%), and other ethnicities [15]. Other studies have also shown a higher proportion of people identified as Hispanics, non-Hispanic, and blacks to present with confirmed cases of COVID-19 due to having underlying co-morbidities [28,29,30]. Authors have attributed this to low socioeconomic status and lack of access to health care disparities among the non-white races. 

The ACE2 (angiotensin-converting enzyme-2) receptors expressed by the epithelial cells of the lungs, intestine, kidneys, and blood vessels have been identified as a target receptor for coronaviruses [31]. Patients with hypertension and diabetes treated with ACE inhibitors show increased expression of ACE2 [32]. The immunosuppressive effects of hyperglycemia could also explain why patients that develop acute respiratory distress syndrome (ARDS) due to COVID-19 were found to have statistically significant elevated glucose levels in the blood. This finding has important implications given the high global prevalence of diabetes [33]. 

The poor outcomes for COVID 19 related to the cardiovascular co-morbidities may be a direct result of the condition itself or attributed to other conditions [34]. Some authors have found rather modest associations of adverse outcomes and hypertensive comorbidities like Atkins JL et.al. with respect to the UK cohort [35]. Our study lacks stratification of comorbidities according to complications of hypertension, obesity, diabetes, and dementia.

Obesity was the significant comorbidity seen in the COVID-19 patients in our study. Obesity is associated with altered pulmonary function, it causes a decrease in the respiratory system compliance, expiratory reserve volume, functional capacity. Obesity is also associated with increased cytokines which may contribute to the increased risk of severe COVID-19 infection [36,37,38,39]. This is in concordance with previous studies also finding independent associations of body mass index (BMI) with outcomes in COVID-19 patients irrespective of the status of obesity. This can be because increased ectopic fat distribution including visceral, perivascular, and epicardial distribution promotes chronic pro-inflammatory, prothrombotic and vasoconstrictive states manifesting as insulin resistance, type 2 diabetes, hypertension, atherosclerosis, immunocompromised state, and cardiovascular diseases [33,35,40,41].

With almost all the patients suspected to have COVID-19 before the definite diagnosis being established, a variety of atypical symptoms could be documented. Those patients who reported specific symptoms like cough and shortness of breath had significantly higher chances of contracting severe SARS-CoV-2 infection and subsequently the need for intubation. However, some atypical symptoms like diarrhea and fatigue also had significantly fatal outcomes. This may have clinical implications in predicting the severity, need for intubation, and mortality even at the very first presentation of a patient with high suspicion of being suffering from COVID-19.

Our study reported that 96.5% had a history of exposure to SARS-CoV-2 and only 3.5% of patients reported not having been exposed to or been in close contact with a known case of COVID-19 before the onset of symptoms. Such findings have sometimes been attributed to the characteristic superspreading events reported in connection with the severe acute respiratory syndrome coronavirus (SARS-CoV) and Middle East respiratory syndrome coronavirus (MERS-CoV) infections. For SARS-CoV-2, the degree to which superspreading is involved in transmission remains unclear, but there is growing evidence that SSEs might be a typical feature of COVID-19 [42,43].

The most common treatment provided to patients in our study was azithromycin. Azithromycin is directed against the virus, whereas Hydroxychloroquine (HCQ) is directed against cellular adhesion cofactors. A study by Fantini et al. indicates that Chloroquine and Azithromycin act via mirror competitive mechanisms. The virus binds to lipid raft through spike protein which has a ganglioside binding domain. This domain can be occupied by GM1 sugar sharing molecular similarity with azithromycin. While azithromycin does this, chloroquine covers the ganglioside surface complementing it. The concerns arising due to their structural similarity, which may reduce the potential effectiveness, have been noticeable as they both synergistically decrease the SARS-CoV-2 load in infected patients [44]. Binding to these two sites can lead to a synergistic antiviral mechanism at the membrane level [45]. Furthermore, azithromycin reduces superadded bacterial infection in these patients, leading to less severe disease [45,46,47,48,49,50,51]. By analyzing the use of drugs such as Remdesivir and Tocilizumab, we found that though these drugs were used significantly more in patients with severe COVID. However, they were significantly associated with worse clinical outcomes which imply that both these drugs failed to provide any survival benefit to these patients. This is in concordance with the recent studies demonstrating that Remdesivir only shortened hospital stay when used in the early days of the infection and did not improve mortality in patients with severe COVID-19 infection [52].

The length of stay (LOS) remains an important but underreported characteristic parameter that has both clinical and public health implications. Our study found that the average length of stay was just under 3 days for people suffering from COVID-19, which may have been skewed due to the data representing the majority of asymptomatic and non-severe patients.

When the LOS was stratified according to the initial presenting symptoms, the highest LOS was associated with patients presenting with fatigue (6.2 days) and shortness of breath (5.8 days), probably because fatigue was significantly associated with mortality and shortness of breath was associated with higher severity and intubation. This was followed by the LOS of respiratory distress (5.5 days), fever (4 days), and cough (2.7 days). A similar analysis by Wu S et.al. found strong evidence to indicate that patients having fever before admission had significantly longer LOS than those without fever by 3.5 days (95% CI 1.39 to 5.63, *p* = 0.002), which was similar to what we found in association of fever, but per analysis suggested shortness of breath and fatigue to be associated with higher LOS with similar significance levels of *p*-value [53]. This data can be especially important for predicting hospital bed requirements in public health response. Better predictions can be made by associating the comorbidities of patients with the length of stay in both symptomatic and asymptomatic patients [53,54,55]. 

With help of multiple logistic regression models, the severity of disease according to the parameters of our study showed that higher age, male sex, and symptoms like shortness of breath and cough, presence of comorbidities particularly hypertension, diabetes, and obesity, and need for treatment were independent significant predictors. Whereas age, symptoms like shortness of breath, fatigue, and diarrhea along with the presence of comorbidities including hypertension symptomatic infection, and need for treatment were independent significant predictors of mortality. 

These results have implications for both preventive and curative interventions for a targeted prioritization especially in resource-poor settings which may prove better than using broad spectrums of comorbidities like age as a blanket risk factor for severity [56,57]. 

In this study, the total number of deaths observed was 84 (4.8%) with the majority of the deaths in hospitalized patients (18.96%). The in-hospital mortality in the current study was similar to that reported in other published US studies (15.3–24.5%) [26,58]. Only 1.08% of the deaths were out of hospital inpatients discharged to nursing homes and from ER. Mortality increased in association with increasing age as expected. Hypertension was also associated with increased mortality but did not reach statistical significance. 

There were several limitations in our study. First, our study was retrospective in nature and solely relied on medical records, due to which we could not collect the data that was missing in the medical records. Second, the COVID-19 cases included in our study were those presenting to the hospitals in a single university system, which may not be widely representative of other scenarios. Third, our study sample was not diverse enough, due to the demographic distribution of the majority of the White population in West Virginia, and thus we could not evaluate further associations by race/ethnicity due to smaller samples of minority populations. Fourth, we did not have enough data to accurately define the event for survival analysis. Hence, we proposed our definition of event based on severity and death variables. 

## 5. Conclusions

This single-center study of COVID-19 patients from West Virginia State Hospitals showed that the older age (≥50 years) and presence of comorbidities (hypertension, diabetes, and obesity) are the most important predictors of outcome in COVID-19 infection. Shortness of breath was the most significant symptom associated with the severity, mortality, and prolonged hospital stay. 

## Figures and Tables

**Table 1:** Clinical characteristics, treatments, and outcomes of patients attending hospitals affiliated with West Virginia University (*n* = 1742).

Characteristics	Yes	No
	*n* (%)	*n* (%)
**Symptoms**		
Cough	72 (4.1)	1670 (95.9)
Cold/flu	27 (1.5)	1715 (98.5)
Shortness of breath	204 (11.7)	1538 (88.3)
Respiratory distress	2 (0.1)	1740 (99.9)
Fever	68 (3.9)	1674 (96.1)
Sore throat	10 (0.6)	1732 (99.4)
Headache	11 (0.6)	1731 (99.4)
Fatigue	41 (2.4)	1701 (97.6)
Body ache	13 (0.7)	1729 (99.3)
Nausea/vomiting	30 (1.7)	1712 (98.3)
Diarrhea	6 (0.3)	1736 (99.7)
**Comorbidities**		
Hypertension	792 (45.5)	950 (54.5)
Diabetes	436 (25)	1306 (75)
Obesity	441 (25.3)	1301 (74.7)
**Treatment**		
Azithromycin	157 (9)	1585 (91)
Hydroxychloroquine	58 (3.3)	1684 (96.7)
Remdesivir	32 (1.8)	1710 (98.2)
Hydroxychloroquine + azithromycin	46 (2.8)	1621 (97.2)
Tocilizumab	28 (1.6)	1714 (98.4)
Vasopressor (septic shock)	37 (2.1)	1705 (97.9)
Intubation	124 (7.1)	1618 (92.9)
**Outcome**		
Severe	131 (7.5)	1611 (92.5)
Total deaths	84 (4.8)	1658 (95.2)
Inpatient deaths	69 (18.96)	295 (81.04)
Out of hospital deaths	15 (1.08)	1363 (98.93)

**Table 2:** Intubation, severity, and outcomes for different characteristics.

Characteristics	No Intubation	Intubation	Fisher Test (*p*-Value)	Not Dead	Dead	Fisher Test (*p*-Value)	Not Severe	Severe	Fisher Test (*p*-Value)
	*n* (%)	*n* (%)		*n* (%)	*n* (%)		*n* (%)	*n* (%)	
**Age (in Years)**									
<50	891 (98)	22 (2)	**<0.001**	908 (99)	5 (1)	<0.001	891 (98)	22 (2)	**<0.001**
≥50	727 (88)	102 (12)		750 (90)	79 (10)		720 (87)	109 (13)	
Sex									
Male	732 (91)	69 (9)	**0.031**	758 (95)	43 (5)	0.37	728 (91)	73 (9)	**0.023**
Female	886 (94)	55 (6)		900 (96)	41 (4)		883 (94)	58 (6)	
Race									
Whites	1360 (92)	112 (8)	0.243	1394 (95)	78 (5)	0.051	1353 (92)	119 (8)	0.198
Others	185 (95)	10 (5)		191 (98)	4 (2)		185 (95)	10 (5)	
Cough									
No	1555 (93)	115 (7)	0.095	1591 (95)	79 (5)	0.391	1550 (93)	120 (7)	**0.019**
Yes	63 (88)	9 (12)		67 (93)	5 (7)		61 (85)	11 (15)	
Cold/flu									
No	1594 (93)	121 (7)	0.435	1633 (95)	82 (5)	0.377	1587 (93)	128 (7)	0.451
Yes	24 (89)	3 (11)		25 (93)	2 (7)		24 (89)	3 (11)	
Shortness of breath									
No	1445 (94)	93 (6)	**<0.001**	1472 (96)	66 (4)	**0.008**	1438 (93)	100 (7)	**<0.001**
Yes	173 (85)	31 (15)		186 (91)	18 (9)		173 (85)	31 (15)	
Respiratory distress									
No	1616 (93)	124 (7)	1	1657 (95)	83 (5)	0.094	1609 (92)	131 (8)	1
Yes	2 (100)	0 (0)		1 (50)	1 (50)		2 (100)	0 (0)	
Fever									
No	1556 (93)	118 (7)	0.627	1596 (95)	78 (5)	0.138	1549 (93)	125 (7)	0.638
Yes	62 (91)	6 (9)		62 (91)	6 (9)		62 (91)	6 (9)	
Sore throat									
No	1608 (93)	124 (7)	1	1648 (95)	84 (5)	1	1601 (92)	131 (8)	1
Yes	10 (100)	0 (0)		10 (100)	0 (0)		10 (100)	0 (0)	
Headache									
No	1607 (93)	124 (7)	1	1647 (95)	84 (5)	1	1600 (92)	131 (8)	1
Yes	11 (100)	0 (0)		11 (100)	0 (0)		11 (100)	0 (0)	
Fatigue									
No	1583 (93)	118 (7)	0.066	1622 (95)	79 (5)	**0.044**	1576 (93)	125 (7)	0.121
Yes	35 (85)	6 (15)		36 (88)	5 (12)		35 (85)	6 (15)	
Body ache									
No	1606 (93)	123 (7)	1	1646 (95)	83 (5)	0.475	1599 (92)	130 (8)	1
Yes	12 (92)	1 (8)		12 (92)	1 (8)		12 (92)	1 (8)	
Nausea/vomiting									
No	1590 (93)	122 (7)	1	1630 (95)	82 (5)	0.653	1583 (92)	129 (8)	1
Yes	28 (93)	2 (7)		28 (93)	2 (7)		28 (93)	2 (7)	
Diarrhea									
No	1613 (93)	123 (7)	0.358	1654 (95)	82 (5)	**0.03**	1606 (93)	130 (7)	0.375
Yes	5 (83)	1 (17)		4 (67)	2 (33)		5 (83)	1 (17)	
Hypertension									
No	937 (99)	13 (1)	**<0.001**	943 (99)	7 (1)	**<0.001**	934 (98)	16 (2)	**<0.001**
Yes	681 (86)	111 (14)		715 (90)	77 (10)		677 (85)	115 (15)	
Diabetes									
No	1257 (96)	49 (4)	**<0.001**	1272 (97)	34 (3)	**<0.001**	1255 (96)	51 (4)	**<0.001**
Yes	361 (83)	75 (17)		386 (89)	50 (11)		356 (82)	80 (18)	
Obesity									
No	1243 (96)	58 (4)	**<0.001**	1246 (96)	55 (4)	**0.053**	1238 (95)	63 (5)	**<0.001**
Yes	375 (85)	66 (15)		412 (93)	29 (7)		373 (85)	68 (15)	

Bold indicates significant *p*-values (*p* < 0.05).

**Table 3:** Length of hospital stay (in days) in the first visit.

Symptoms	With Symptom			Without Symptom			Wilcoxon Test (*p*-Value)
	N1	Mean	SD	N2	Mean	SD	
Cough	72	2.736	4.744	1670	2.203	7.063	**<0.001**
Cold/flu	27	2.185	4.915	1715	2.226	7.011	0.372
Shortness of breath	204	5.779	9.686	1538	1.754	6.397	**<0.001**
Respiratory distress	2	5.5	6.364	1740	2.221	6.983	**0.015**
Fever	68	3.956	7.021	1674	2.155	6.973	**<0.001**
Sore throat	10	0	0	1732	2.238	7	0.096
Headache	11	3.636	7.646	1731	2.216	6.979	0.26
Fatigue	41	6.22	7.302	1701	2.129	6.948	**<0.001**
Body ache	13	1	3.606	1729	2.234	7.001	0.249
Nausea/vomiting	30	3.6	5.739	1712	2.201	7.001	**<0.001**
Diarrhea	6	6.333	9.933	1736	2.211	6.969	0.073

Bold indicates significant *p*-values (*p* < 0.05).

**Table 4:** (a) Effects of baseline characteristics on the severity of COVID-19 without and with treatment using multivariable logistic regression. (b) Effects of baseline characteristics on mortality of COVID-19 without and with treatment using multivariable logistic regression.

**(a)**					
**Severity without Treatment as Outcome**					
Outcomes	Variables	Odds ratio	Lower95%CI	Upper95%CI	*p*-value
Severity	Age	1.03	1.02	1.04	<0.0001
	Sex	0.59	0.40	0.87	0.007
	Shortness of breath	1.72	1.08	2.74	0.024
	Hypertension	2.56	1.35	4.84	0.004
	Diabetes	1.85	1.22	2.83	0.004
	Obesity	2.07	1.38	3.11	0.0005
**Severity with Treatment as Outcome**					
Outcomes	Variables	Odds ratio	Lower95%CI	Upper95%CI	*p*-value
Severity	Age	1.03	1.01	1.04	<0.0001
	Sex	0.59	0.40	0.90	0.02
	Shortness of breath	1.14	0.67	1.94	0.62
	Hypertension	2.24	1.16	4.33	0.02
	Diabetes	1.92	1.22	3.01	0.005
	Obesity	1.92	1.21	2.91	0.004
	Azithromycin	1.99	1.14	3.45	0.02
	Hydroxychloroquine	1.71	0.79	3.72	0.17
	Remdesivir	2.84	1.18	6.82	0.02
	Tocilizumab	10.39	3.94	27.24	<0.0001
**(b)**					
**Mortality without Treatment as Outcome**					
Outcomes	Variables	Odds ratio	Lower95%CI	Upper95%CI	*p*-value
Mortality	Age	1.07	1.05	1.08	<0.0001
	Shortness of breath	1.67	0.92	3.01	0.09
	Diarrhea	8.63	0.82	91.08	0.07
	Hypertension	2.83	1.19	6.72	0.02
	Diabetes	1.58	0.96	2.60	0.07
**Mortality with Treatment as Outcome**					
Outcomes	Variables	Odds ratio	Lower95%CI	Upper95%CI	*p*-value
Mortality	Age	1.06	1.05	1.08	<0.0001
	Shortness of breath	1.36	0.72	2.53	0.34
	Diarrhea	6.25	0.75	51.76	0.09
	Hypertension	2.99	1.30	6.97	0.01
	Azithromycin	1.72	0.90	3.29	0.12
	Hydroxychloroquine	1.69	0.70	4.14	0.25
	Remdesivir	4.24	1.71	10.50	0.002
	Tocilizumab	2.85	1.01	8.07	0.05

## Data Availability

Data was extracted from WVU and accessed only by designated personnel at WVU.
